# Perioperative alendronate, risedronate, calcitonin and indomethacin treatment alters femoral stem fixation and periprosthetic bone mineral density in ovariectomized rats

**DOI:** 10.1007/s00776-015-0717-5

**Published:** 2015-03-26

**Authors:** Deniz Cankaya, Yalcin Tabak, Akif Muhtar Ozturk, Muhammed Cuneyd Gunay

**Affiliations:** 1 Department of Orthopaedic and Traumatology, Ankara Numune Training and Research Hospital, 06100 Altindag, Ankara Turkey; 2 Gazi University, Ankara, Turkey

## Abstract

**Background:**

Many factors affect implant stability and periprosthetic bone mineral density (BMD) following total joint arthroplasty. We asked whether perioperative alendronate, risedronate, calcitonin and indomethacine administration altered (1) femoral stem shear strength and periprosthetic bone mineral density BMD in ovariectomized rats and (2) whether there were differences in the effect of these drugs.

**Methods:**

Thirty overiectomized rats were divided into five groups and implanted with intramedullary mini-cortical screws in the femur. Four groups were treated with alendronate, risedronate, salmon calcitonin and indomethacin for 4 weeks preoperatively and 8 weeks postoperatively.

**Results:**

Although alendronate and risedronate increased the periprosthetic BMD more than calcitonin, they did not alter implant fixation compared to calcitonin. Indomethacin significantly decreased the BMD around the stem and implant stability compared to all other groups.

**Conclusions:**

This study showed that perioperative treatment with bisphosphonates and calcitonin improved the BMD around the stems and implant stability. Although bisphosphonates increased the BMD more than calcitonin, there was no difference in implant stability. Indomethacin markedly decreased the periprosthetic BMD and implant stability. The main clinical significance of our study was the finding about the need to strictly avoid long-term use of high-dose nonsteroidal antiinflammatory drugs for patients who have major joint arthritis and a previous history of arthroplasty.

## Introduction

Recent improvements in prosthesis design have encouraged surgeons to prefer cementless total hip arthroplasty (THA), even for elderly and osteoporotic patients [1]. Due to the poor bone quantity and density in osteoporotic patients, bone growth around the stem is insufficient compared to non-osteoporotic patients [2]. Adequate bone integration, reducing region-specific decreases in femoral bone density and early stability of implant fixation decrease the risk of early migration and loosening and then may reduce late loosening rates [1, 3].

Bisphosphonates and salmon calcitonin are well-known and effective inhibitors of bone resorption. Recent studies have shown that alendronate and risedronate significantly inhibit the decrease of periprosthetic bone mineral density (BMD) and bone resorption in the proximal femur after cementless THA [4–6]. Calcitonin has also effectively enhanced the volume of the bone mass surrounding the prosthesis and also significantly increased the osseointegration rate after THA [7, 8].

Nonsteroidal antiinflammatory drugs (NSAIDs) are widely used analgesic agents for pain control in osteoarthritis, and they also play a major role in postoperative pain management in orthopedic surgery [9]. Indomethacin is one of the representative members of NSAIDs, has a well-known effect on bone formation and drastic inhibitory effects on fracture healing [9, 10]. Despite these facts, the effects of NSAIDs on implant stability and BMD around the stem after THA have not been widely investigated. The research mainly focused on the effect of NSAIDs on BMD values after long-term treatment and fracture healing [9–11].

Although these drugs are widely used in elderly arthroplasty patients alone or together, there has been no general overview study about these drugs. The aim of this study was to analyze and compare the effects of perioperative treatment with alendronate, risedronate, salmon calcitonin and indomethacin on periprosthetic BMD and fixation of the femoral stem in an ovariectomized rat model of osteoporosis; a group without any medical treatment was set as the control group.

## Materials and methods

Thirty rats underwent bilateral ovariectomy 16 weeks before implantation and were randomly divided into five groups with each group comprising six rats. All were Wistar rats (260–280 g) and 24 weeks old as this age group has been shown to have the best osteoporotic response after overiectomy [12]. All rats were housed individually at 22 °C with a 12-h light: 12-h dark cycle in the Animal Research Facilities. The research procedures were in full compliance with Veterinary Medicine Deontology Regulation 6.7.26 and the Helsinki Declaration of Animal Rights and had the approval of the Ethics Committee for Animal Research.

The first group of rats was assigned as the control group, and no other treatment was given to this group before and after implantation. The remaining four groups of ovariectomized rats were treated with their assigned group protocol for 12 weeks, starting 4 weeks before the implantation and continuing for 8 weeks after the implantation. They were given: (1) [ALN] 0.2 mg/kg of alendronate, (2) [RSN] 0.1 mg/kg of risedronate, (3) [CT] 2 IU/kg salmon calcitonin and (4) [IND] 4 mg/kg of indomethacin (Table 1). The ALN drug dosage was 0.1 mg/kg/day in a previous study with a similar experimental model [1]; we assumed the same 0.1 mg/kg as the daily dosage. This was 1 % of the therapeutic drug dose in humans. Therefore, we also used 1 % of the human therapeutic drug dose to determine the drug doses in rats for other drugs. All drugs were diluted with saline solution and applied subcutaneously once every 2 days as double the daily dosage.

**Table 1:** Treatment schedule of the study with 24-week-old Wistar rats (260–280 g)

	Number of rats	Preoperative medication (4 weeks)	Implant	Postoperative medication (8 weeks)
1. Group (control)	6	–	+	–
2. Group (alendroante)	6	0.2 mg/kg (once every 2 days)	+	0.2 mg/kg (once every 2 days)
3. Group (risedronate)	6	0.1 mg/kg (once every 2 days)	+	0.1 mg/kg (once every 2 days)
4. Group (salmon calcitonin)	6	2 IU/kg (once every 2 days)	+	2 IU/kg (once every 2 days)
5. Group (indomethacine)	6	4 mg/kg (once every 2 days)	+	4 mg/kg (once every 2 days)

**Fig. 1:**
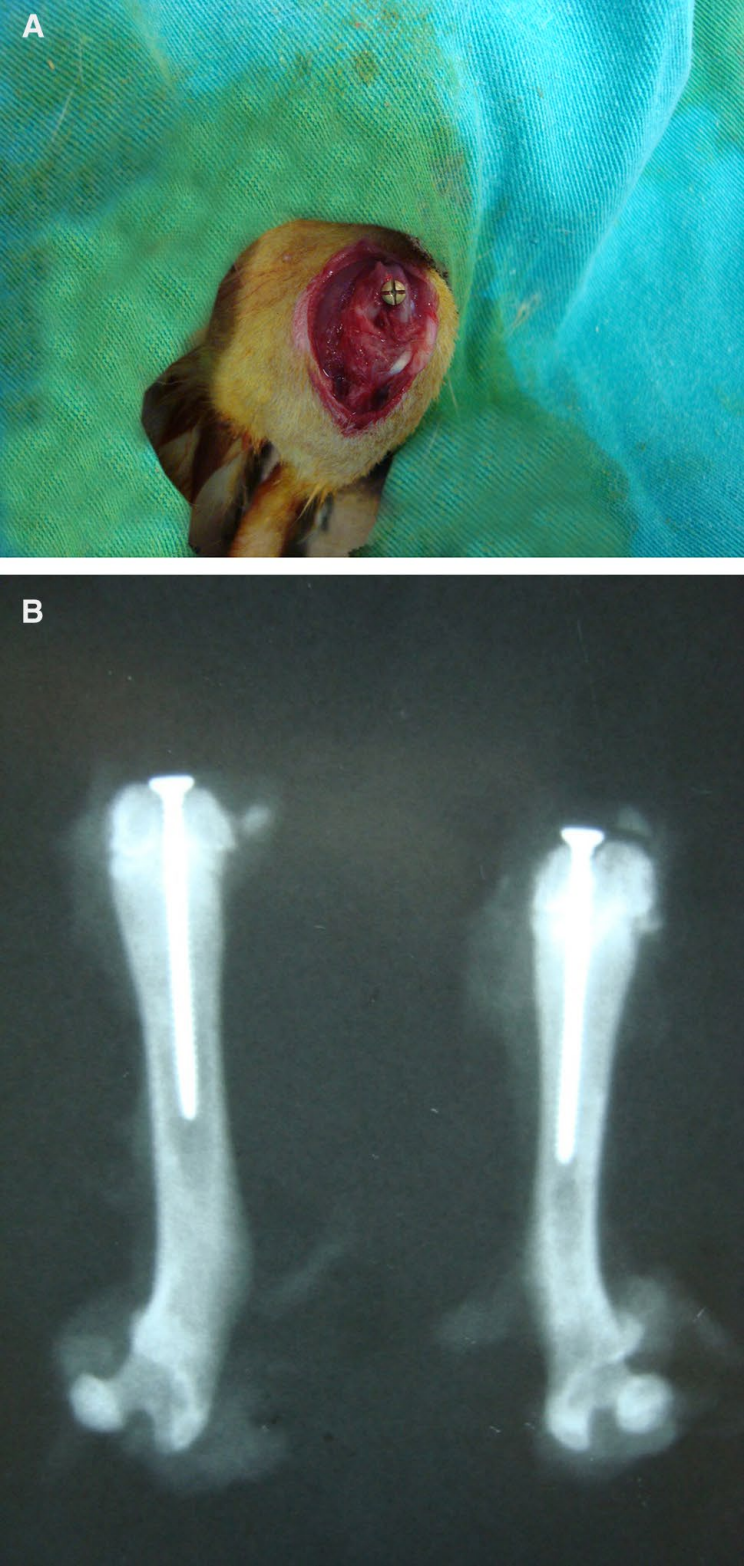
Knee of an overiectomized rat after implantation of a 1.3-mm intramedullary screw (**a**) and X-ray of the implanted femur of the overiectomized rat (**b**)

After 4 weeks, animals were brought to the theatre inside the laboratory and anesthetized with a combination of 45 mg/kg ketamine hydrochloride and 5 mg/kg xylasine hydrochloride. After anesthetic agents had been administered intramuscularly, the surgical area was shaved and washed. A midline skin incision was made in the left knees of rats, and a standard medial parapatellar approach was performed. Using a 1.2-mm drill, the intercondylar notch of the left femur was drilled into the medullary cavity. All implants were 18 mm long and 1.3 mm in diameter (Synthes: 1.3 mm self-taping screw) and implanted into the intramedullary cavity (Fig. 1a). At the end of the operation, the muscle and skins were closed in anatomical layers. There were no wound problems or infections in any of the rats after surgery. Drug application for the groups was continued for a further 8 weeks as mentioned above. All of the rats were killed 8 weeks after implantation with a combination of 60 mg/kg ketamine hydrochloride and 20 mg/kg xylasine hydrochloride given intramuscularly. Implanted femurs of the rats were carefully dissected and excised.

Periprosthetic BMDs of all rats were analyzed by using a Lunar DPX-IQ DXA (NY,USA) using Lunar’s Small Animal Software version 1.0 set in the HiRes (0.6 × 1.2 mm) Medium mode <0.5 kg. All of the dual-energy X-ray absorptiometry (DEXA) measurements were done from the distal metaphyseal region of implanted femurs. The periprosthetic BMD value was calculated as described in a previous study [9]. The BMD value of the screw was measured alone, then the difference between the mean BMD value and BMD value of the screw alone gave us the BMD around the stem. Direct anterior posterior X-rays of the femurs were also taken (Fig. 1b).

Before shear strength measurement, the distal ends of the dissected femurs were trimmed carefully in order to expose the head of the implant. A steel wire was folded in half from both edges. The midportion of the wire was placed under the head of the screw circumferentially and secured there by twisting from both edges. The two opposite free edges of the wires were connected to each other centrally above the head by the longitudinal direction of the screw. The proximal part of the dissected femur was secured inside the hole within the wooden block by using bone cement. The push-out strength was defined as the peak force occurring while the implant loosened from the bone [3]. The shear strength at the bone-implant interface was measured by applying a vertical load to the implant through the wire secured to the head of the screw. Shear strength measurements were done with a Lloyd-LR5 K (Fareham, UK) pressure test device. The pressure test device was used with a constant displacement speed of 0.5 mm/min to measure the shear strength.

The present study was approved by the Animal Experiments Local Ethics Committee prior to performing the study. Statistical analyses were performed with SPSS 13.0 (SPSS Inc., Chicago, IL, USA) and were calculated as the median, mean and standard deviation for all groups. The comparison between groups was done with the Kruskal-Wallis test, and the post hoc test was used to analyze the differences between groups. *p* values <0.05 were considered to indicate a statistically significant difference.

## Results

**Fig. 2:**
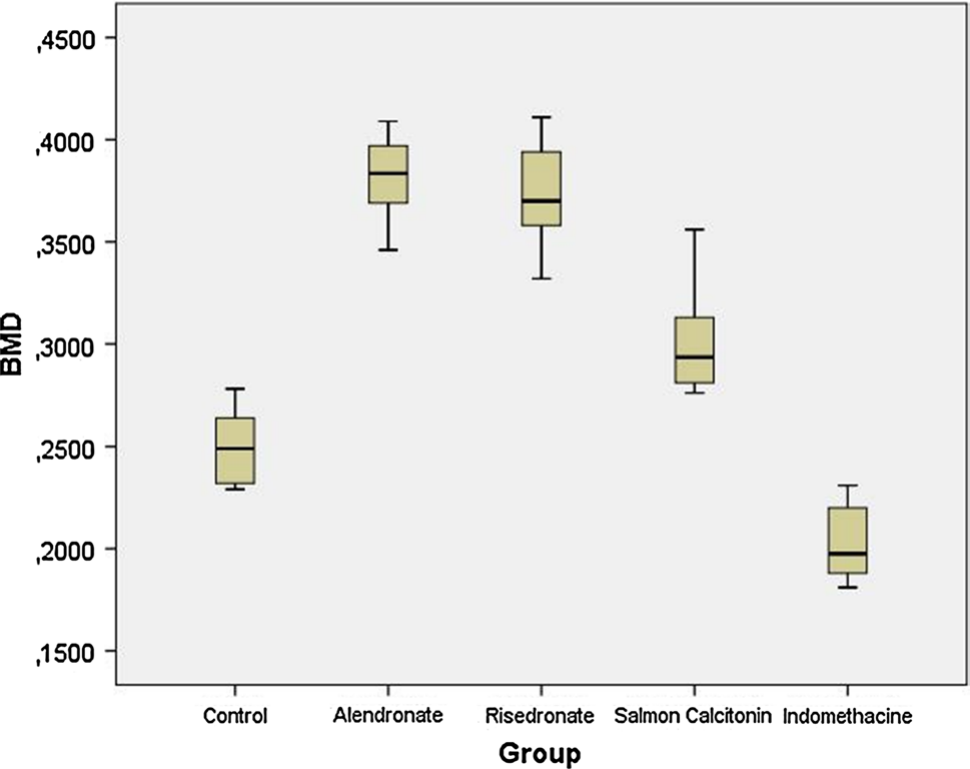
*Boxplots* for comparisons of the periprosthetic bone mineral density (g/cm^2^) of the groups after 12 weeks (4 weeks preoperatively, 8 weeks postoperatively) of treatment. The *line* in *the middle of the box* represents the median for each group

Periprosthetic BMD and bone-implant shear strength results of the groups were calculated as the median, mean and standard deviation and are shown in Table 2. The bone mineral density of the ALN and RSN groups was significantly higher than in the control group (*p* < 0.001). There was no significant difference between these two bisphosphonates in terms of the periprosthetic BMD values of the ALN and RSN groups (*p* > 0.05). The median periprosthetic BMD value of the ALN group was about 35 % higher than that in the control group (0.249 g/cm^2^). Compared with the control group, the CT group had significantly higher periprosthetic BMD values (*p* < 0.01). The periprosthetic BMD values of the ALN and RSN groups were significantly greater than in the CT group (*p* < 0.001), and the median BMD value of this group was about 23 % lower than that in the ALN group. IND significantly decreased the periprosthetic BMD compared to the control (*p* < 0.01) and all other drug groups. The median periprosthetic BMD value of the IND group was about 20 % lower than in the control group. Boxplots for comparisons of the periprosthetic BMDs of the groups are shown in Fig. 2.

**Table 2:** Periprosthetic bone mineral density and bone-implant shear strength of the groups with the mean and median values after 12 weeks (4 weeks before and 8 weeks after implantation) treatment

	Bone mineral density (g/cm^2^)		Bone-implant shear strength (*N*)	
	Mean	Median	Mean	Median
Control	0.250 ± 0.019	0.249 (0.229–0.278)	6.99 ± 0.76	6.98 (5.89–8.11)
Alendronate	0.381 ± 0.022	0.384 (0.346–0.409)	9.65 ± 1.07	9.77 (8.12–11.23)
Risedronate	0.372 ± 0.028	0.370 (0.332–0.411)	9.69 ± 1.13	9.84 (8.02–11.23)
Salmon calcitonin	0.302 ± 0.030	0.294 (0.276–0.356)	9.27 ± 1.15	9.00 (7.71–10.98)
Indomethacine	0.203 ± 0.019	0.198 (0.181–0.231)	5.14 ± 0.69	5.17 (4.12–6.13)

The values are given as the mean ± SD and the median with the minimum-maximum values in parentheses

**Fig. 3:**
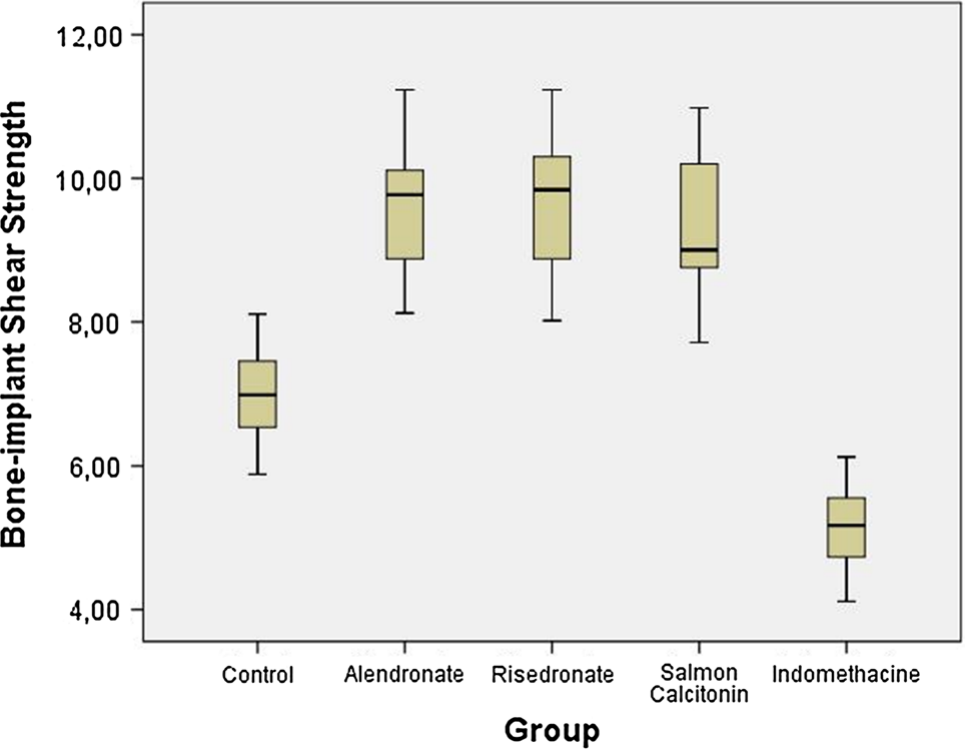
*Boxplots* for comparisons of the bone-implant shear strength* (N*) of the groups after 12 weeks (4 weeks preoperatively, 8 weeks postoperatively) of treatment. The *line in the middle of the box* represents the median for each group

Compared with the control group, the ALN and RSN groups had significantly higher bone-implant shear strength (*p* < 0.001) and were about 29 % higher than in the control group. There was no significant difference between these two bisphosphonates (*p* > 0.05). The bone-implant shear strength of the salmon calcitonin group was significantly greater than in the control group (*p* < 0.001). Although these two bisphosphanates had higher periprosthetic BMD values compared to the salmon calcitonin, regarding the bone-implant shear strength, there was no statistical difference between these two bisphosphonates and salmon calcitonin (*p* > 0.05). Regarding IND, it significantly decreased bone-implant shear strength compared to the control (*p* < 0.01) and all other drug groups (*p* < 0.001). The median value of the indomethacin group was about 26 % lower than that in the control group. Boxplots for comparisons of the bone-implant shear strength of the groups are shown in Fig. 3.

## Discussion

In the present study, we aimed to investigate the effects of clinically well-known anti-osteolytic agents on the integrity of the implant-bone interface. A well-known analgesic drug with established anti-osteoblastic activity was also added to the study design for both observations of its effects on the shear strength and BMD around the implant. The current study clearly demonstrates the additive effect of the bisphosphonates studied in the current model on BMD and implant shear strength. The representative NSAID “indomethacin” was also clearly demonstrated to have detrimental effects on BMD and implant shear strength.

Patients who need total hip arthroplasty (THA) are mainly eldery and have coexisting osteoporosis, so they are vulnerable to the occurrence of negative factors leading to persistent pain, which may lead to the need for undesirable revision surgery [7]. After a cementless total hip arthroplasty, the integrity of the implant-bone interface is among the main determining factors of implant survival. Bone resorption around the periprosthetic area and consequent shifting of the implant would be negative factors for the long-term results [1, 7]. Hence, recent research has mostly focused on improving biological fixation for long-term survival of implants by preventing osteolysis and osteoporotic bone loss at the periprosthectic area [1, 5, 6, 8, 13, 14]. In real life, the implant-bone interface occurs in the metaphyseal area filled with cancellous bone. Overiectomized rat models are associated with cancellous bone loss [1, 2]. For this reason, animal study models based on ovariectomized rats provide a model similar to a postmenopausal osteoporotic bone host [1, 13, 14]. To replicate the clinical situation, we therefore preferred an overiectomized rat model for our study design, and an implant was also placed in the metaphyseal area.

Clinical and experimental studies have shown that bisphosphonates decrease periprosthetic bone loss [4, 5, 13–15] and increase implant stability [1, 13, 16]. Although risedronate and alendronate were shown to inhibit periprosthetic bone loss around the femoral component and increase implant stability after cementless total hip arthroplasty [4–6, 15, 17], no experimental studies have compared them. There was no statistical difference between their positive outcomes, so we concluded that perioperative use of ALN and RSN improved outcomes after cementless THA without one being superior to the other.

Salmon calcitonin is the other most commonly used antiosteoporotic drug beside bisphosphonates. Several studies showing that calcitonin decreases bone loss after bone fracture and cementless THA [7, 8, 18, 19]. Duarte et al. [19, 20]  demonstrated that CT administration resulted in an increase in bone density around the implant, but this increase was not significant, and ALN therapy might be effective in the prevention of bone loss around implants in overiectomized rats. The present comparative study supported a previous comparative study concluding the effect of ALN on osseointegration of implants is superior to salmon CT [7]. Chen et al. [7] concluded that ALN might be a better choice than salmon CT in order to maintain better stability of the prosthesis because of their histological findings. This suggestion is disputable, as the bone integration at the entire implant could not be measured by histological studies, and these studies provide limited information regarding bone-implant fixation. In the present study, although ALN and RSN had slightly higher mean shear strength compared to salmon CT, no significant difference was detected. Thus, they are both beneficial for osteoporotic patients in need of a total joint arthroplasty, and no one of these two major osteoporosis drug groups is superior regarding the implant stability.

Because of their analgesic and antiinflammatory effects, nonsteroidal antiinflammatory medications are among the most commonly prescribed drugs [21]. They are widely used by patients who have undergone major joint arthroplasties before and after the surgical procedure. However, there have been few mechanical studies on the effect of NSAIDs on implant fixation in experimental animal models, and these studies were mainly done in the 1990s [22–24]. Contradictions in these studies emphasized the necessity of further investigations of NSAIDs [21]. Trancik et al. [23] concluded that IND, aspirin and ibuprofen decreased bone ingrowth in a dose-related fashion up to 8 weeks. IND was shown to decrease the cancellous bone volume in overiectomized rats up to 24 weeks. Dimmen et al. [9] reported a 14 % decrease in periprosthetic BMD in their intramedullary implanted fractured tibia model in rats after 3-week treatment.

However, Cook et al. [22] suggested that perioperative administration of indomethacin did not significantly affect the attachment strength or bone ingrowth into implants in the long-term period. The results of this study were contradictory to our and other studies. In the present study, IND significantly decreased the periprosthetic BMD value and implant stability. In addition to possible reasons for the contradictory results, there are some limitations to this study and to using its results for cementless THA in elderly patients. These include the transverse bicortical placement of the implants, the choice of the cortical diaphyseal region for implantation and lack of overiectomy surgery in the experimental rats.

There are some limitations to this study. First, it was an experimental study, which limits the direct application of the results to clinical practice, like in any other experimental study. Second, there was no histopathological examination because of the detrimental effect of mechanical examination on tissue integrity and the inadequate number of experimental animals. In addition, bisphosphonates are also used with breaks by the patients. As we examined the continuous use of these drugs, the effect of taking breaks in the use of these drugs could not be determined. Finally, the relative shortness of the treatment period in the present study did not provide any information about the effect of these drugs in the long-term period.

In summary, although bisphosphonates increased the BMD around the stem more than salmon calcitonin, there was no difference in implant stability; thus, they are both beneficial for osteoporotic patients in need of total joint arthroplasty. We concluded that clinicians have to strictly avoid the long-term use of high-dose NSAIDs for osteoporotic patients who have major joint arthritis and previous arthroplasty history. Alternative treatment modalities in pain management can be applied in the postoperative period to decrease the usual NSAID dose. However, as data on this issue are limited, further studies with different doses and treatment durations are needed to reach generally accepted conclusions about the use of NSAIDs in arthroplasty patients before and after surgical procedures.
